# Construction and Analysis of the Protein-Protein Interaction Networks Based on Gene Expression Profiles of Parkinson's Disease

**DOI:** 10.1371/journal.pone.0103047

**Published:** 2014-08-29

**Authors:** Hindol Rakshit, Nitin Rathi, Debjani Roy

**Affiliations:** 1 Integrated Science Education & Research Centre (ISERC), Visva-Bharati University, Shantiniketan, Birbhum, West Bengal, India; 2 Cognizant Technology Solutions India Pvt. Ltd., Rajiv Gandhi Infotech Park, MIDC, Hinjewadi, Pune, Maharashtra, India; 3 Department of Biophysics, Bose Institute, Acharya J.C. Bose Centenary Building, Kolkata, West Bengal, India; University of Cincinnati, United States of America

## Abstract

**Background:**

Parkinson's Disease (PD) is one of the most prevailing neurodegenerative diseases. Improving diagnoses and treatments of this disease is essential, as currently there exists no cure for this disease. Microarray and proteomics data have revealed abnormal expression of several genes and proteins responsible for PD. Nevertheless, few studies have been reported involving PD-specific protein-protein interactions.

**Results:**

Microarray based gene expression data and protein-protein interaction (PPI) databases were combined to construct the PPI networks of differentially expressed (DE) genes in post mortem brain tissue samples of patients with Parkinson's disease. Samples were collected from the substantia nigra and the frontal cerebral cortex. From the microarray data, two sets of DE genes were selected by 2-tailed *t*-tests and Significance Analysis of Microarrays (SAM), run separately to construct two Query-Query PPI (QQPPI) networks. Several topological properties of these networks were studied. Nodes with High Connectivity (hubs) and High Betweenness Low Connectivity (bottlenecks) were identified to be the most significant nodes of the networks. Three and four-cliques were identified in the QQPPI networks. These cliques contain most of the topologically significant nodes of the networks which form core functional modules consisting of tightly knitted sub-networks. Hitherto unreported 37 PD disease markers were identified based on their topological significance in the networks. Of these 37 markers, eight were significantly involved in the core functional modules and showed significant change in co-expression levels. Four (ARRB2, STX1A, TFRC and MARCKS) out of the 37 markers were found to be associated with several neurotransmitters including dopamine.

**Conclusion:**

This study represents a novel investigation of the PPI networks for PD, a complex disease. 37 proteins identified in our study can be considered as PD network biomarkers. These network biomarkers may provide as potential therapeutic targets for PD applications development.

## Introduction

Parkinson's disease (PD) is a neurodegenerative disorder of the central nervous system. It is the second most common degenerative disorder after Alzheimer's disease, affecting more than 1% of those over the age of 55 years and more than 3% of those over the age of 75 years [1]. PD is characterized by tremor, muscle rigidity, and slowed movement (bradykinesia). The motor symptoms of PD result from the death of dopamine generating cells in the substantia nigra, a region of the mid brain. Improving diagnoses and treatment of this disease is essential, as currently there exists no cure for PD.

For a long time, PD has been considered to be a non-genetic disorder; however around 15% of patients with PD are known to have a first-degree relative who is also affected by this disease [2]. Mutations in several specific genes have been conclusively shown to be associated with PD. These genes code for alpha-synuclein (SNCA), parkin (PRKN), leucine-rich repeat kinase 2 (LRRK2 or dardarin), PTEN-induced putative kinase 1 (PINK1), DJ-1 and ATP13A2 [3], [4]. The most extensively studied PD-related genes are SNCA and LRRK2 [1]. Mutations in SNCA, LRRK2 and glucocerebrosidase (GBA) are associated with most of the PD related cases [1]. Nevertheless, very less amount of work has been done related to protein interactions specific to the disease state.

Network science is gradually altering our view of cell biology by offering unforeseen possibilities to understand the internal organization of a cell [5]. The developments of high-throughput data-collection techniques have brought insights to our understanding of diseases. Sincere amount of time and effort has to be devoted in order to analyse this vast amount of data if we want to understand the interrelationships among disease-related genes and proteins [5]. In 2009, Taylor *et al.* [6] studied gene expression based weighted Protein-Protein Interaction (PPI) networks for breast cancer. They found that loss of gene co-expression of proteins interacting within the BRCA1-associated genome surveillance complex (BASC) is associated with poor outcomes of the disease. In 2011, Lee *et al.* [7] constructed protein-protein interaction (PPI) networks of abnormally expressed genes for schizophrenia, bipolar disease and major depression, and identified several disease markers like SBNO2 for schizophrenia, SEC24C for bipolar disorder, and SRRT for major depression. Recently, in April 2013, Ran *et al.* [8] constructed and analysed PPI networks for Essential Hypertension (EH), and suggested that blood pressure variation related to EH is orchestrated by an integrated PPI network with the protein encoded by NOS3 gene as its backbone.

In this study, PPI networks were constructed for PD using proteins which code for differentially expressed genes only in substantia nigra and frontal cerebral cortex. The PPI networks were constructed based on the following assumptions [7]

1. Expression level of most of the proteins and mRNAs in the brain are positively correlated.
2. Proteins with similar expression patterns are more likely to interact with each other.
3. Abundant proteins participate more in biological processes.

Topological analyses were performed to find out the significant network biomarkers. The association of these biomarkers with PD-related genes and neurotransmitters were studied. Several complexes were also studied in the networks. Changes of co-expression level of genes associated with the complexes from control to disease state were also studied. 37 unreported disease marker genes were identified of which eight were significantly involved in the core functional modules and four showed strong association with several neurotransmitters, including dopamine. Thus our study may provide insights into the potential targets for developing new treatments for PD.

## Methods

### Sources of microarray data

**Figure 1:**
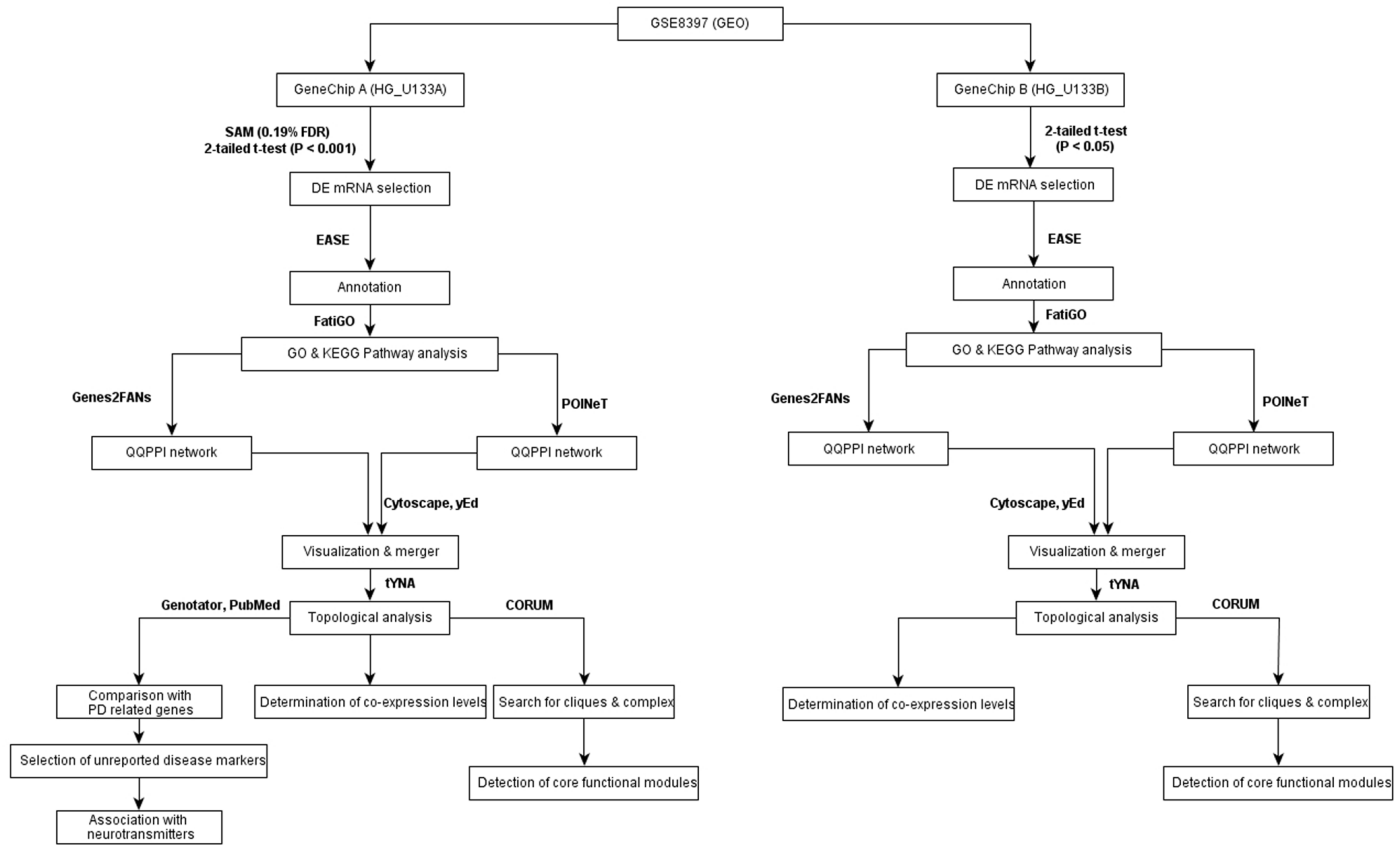
Research methodology.

**Figure 1** gives the flowchart of research methodology applied in this study. The raw data (CEL files) of microarray data series GSE8397 were downloaded from Gene Expression Omnibus (GEO) (http://www.ncbi.nlm.nih.gov/geo/) and normalized by gcRMA [9]. GSE8397 was published by Moran et al. in 2006 [10]. It contains 47 individual localized brain tissue samples of the substantia nigra (SN) (split into medial and lateral portions) and frontal cerebral cortex (FCC) associated with PD as well as control cases, using A (HG_U133A) and B (HG_U133B) Gene Chip per sample. 15 samples of medial parkinsonian SN (MSN), 9 samples of lateral parkinsonian SN (LSN) and 5 samples of parkinsonian FCC were taken. 8 MSN samples, 7 LSN samples and 3 FCC control samples were considered.

Our protein-interaction networks were built based on differentially expressed genes of MSN and LSN only. Initially we started a region wise study of three parts of the brain viz., MSN, LSN and FCC. When we performed 2-tailed t-test and SAM, we did not get any differentially expressed genes for FCC. MSN and LSN separately yielded less number of differentially expressed genes. However, when we combined both MSN and LSN, it yielded significant number of differentially expressed genes. Therefore the data presented in our manuscript is the collection of genes present in combined MSN and LSN.

### Selection of differentially expressed genes, annotation & gene ontology (GO) analysis

Both 2-tailed *t*-test [7] and SAM [11] were used separately to obtain all possible differentially expressed genes from the microarray data. Expression Analysis Systematic Explorer (EASE) [12] was used to convert the Affymetrix probe IDs into gene symbols. A particular module in Babelomics 4.3.0 [13], FatiGO (http://www.fatigo.org/) [14], was used to extract relevant GO terms for a group of genes with respect to rest of the genes. FatiGO was used to find the over-representative biological processes, molecular functions, cellular components and KEGG pathways [15] involving the DE genes (*p*-value<0.05) (**Table 1**). Among the GO terms, DE genes were most abundant in the over-representative biological processes. These DE genes were considered as the most significant genes in the dataset, and therefore subjected for network construction.

**Table 1:** Gene Ontology (GO) analysis of DE genes.

GeneChip A (HG_133A)								
GO terms →	Cellular Component		Biological Process		Molecular Function		KEGG	
	Terms	Involved Genes	Terms	Involved Genes	Terms	Involved Genes	Terms	Involved Genes
**Dataset obtained by 2-tailed ** ***t*** **-test (P<0.001)**	120	642	792	779	183	677	60	277
**Dataset obtained by SAM (FDR 0.19%)**	67	160	381	207	97	187	36	219
**Common**	33	49	251	58	47	52	19	53

GeneChip B (HG_133B)								
**Dataset obtained by 2-tailed ** ***t*** **-test (P<0.05)**	28	142	61	221	24	107	18	91

For the sake of clarity, we have denoted the set of significant DE genes extracted from GeneChip A using 2-tailed *t*-test, by the symbol 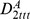, the set of significant DE genes extracted from GeneChip A using SAM, by the symbol 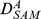, and the set of significant DE genes extracted from GeneChip B using 2-tailed *t*-test, by the symbol 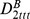. These sets of significant DE genes (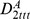, 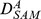 & 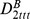) were subjected for construction of protein-protein interaction (PPI) networks.

### Construction of the QQPPI networks

**Figure 2:**
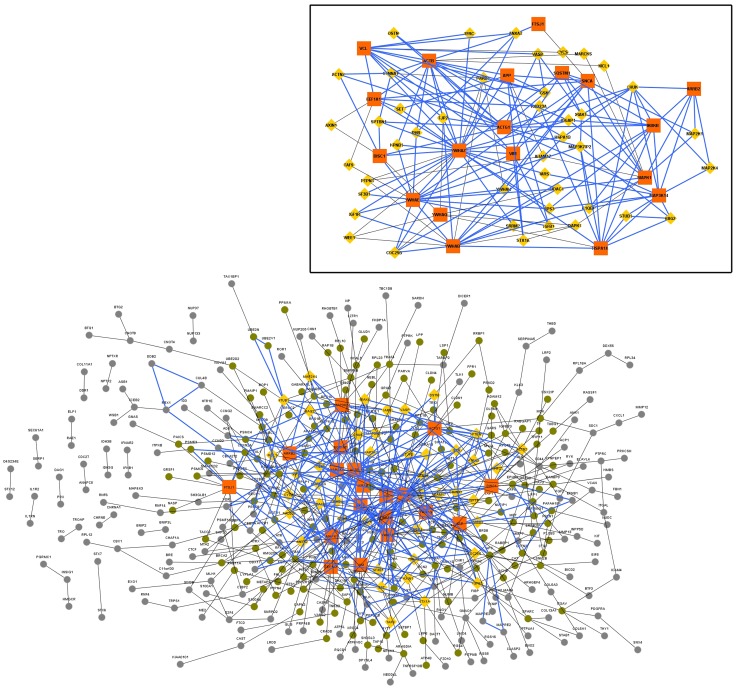
QQPPI network built from the dataset obtained using t-tailed *t*-test (P<0.001) (GeneChip A). Orange coloured square nodes represent hubs (HC nodes). Yellow coloured triangular nodes represent bottlenecks (bottlenecks). The core functional module containing 3,4-cliques are represented using blue coloured edges. Non-hub non-bottleneck nodes are coloured green if they are directly connected to a hub or a bottleneck, and grey otherwise. Inset: Subset of the QQPPI network containing hubs and bottlenecks only.

Two separate approaches were taken to construct the PPI networks. First, Genes2FANs (http://actin.pharm.mssm.edu/genes2FANs/) [16] was used to construct a Query-Query PPI (QQPPI) network, i.e., a network of protein-protein interactions consisting of query nodes only. Secondly, brain tissue specific and experimentally verified data was taken from POINeT (http://poinet.bioinformatics.tw/) [17] to create another QQPPI network. The two networks constructed by Genes2FANs and POINeT were separately viewed using the open source network visualization software Cytoscape 2.8.0 (http://www.cytoscape.org/) [18]. The two networks (developed by Genes2FANs and POINeT) were then merged to construct the final QQPPI network, which includes all the interactions present in both the individual networks. This final network was formatted and visualized using the graph editing software yEd (http://www.yworks.com/) [19]. The same procedure was repeated for the datasets 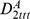, 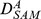 and 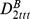. For the sake of clarity, we denote the merged QQPPI network formed by 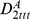 as 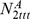, the merged QQPPI network formed by 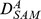 as 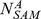, and the merged QQPPI network formed by 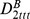 as 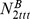 (**Figure 2** **,** **3** **, S1**). Here this must be remembered that the algorithm for QQPPI network is built in such a way that a protein occurs only once in each of the networks.

**Figure 3:**
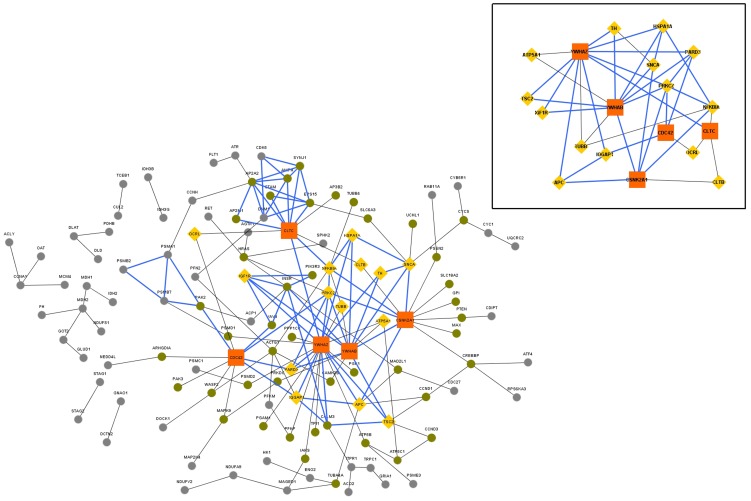
QQPPI network built from the dataset obtained using SAM (FDR 0.19%) (GeneChip A). Orange coloured square nodes represent hubs (HC nodes). Yellow coloured triangular nodes represent bottlenecks (bottlenecks). The core functional module containing 3,4-cliques are represented using blue coloured edges. Non-hub non-bottleneck nodes are coloured green if they are directly connected to a hub or a bottleneck, and grey otherwise. Inset: Subset of the QQPPI network containing hubs and bottlenecks only.

### Topological parameters of QQPPI networks

We analysed topological properties of these networks using the tYNA (http://tyna.gersteinlab.org/) [20] web interface. Global properties of the networks are given in **Table 2**. The topologically significant nodes were extracted from the networks in two steps:

**Table 2:** Global properties of the networks.

GeneChip A (HG_133A)						
Network source	Number of nodes	Number of edges	Average degree	Highest degree	Average betweenness	Highest betweenness
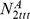	406	690	3.4	47	583.42	21,286.48
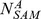	121	172	2.8	21	115.23	1888.753876

1. In the networks, nodes with degree greater than or equal to the sum of mean and twice the standard deviation (S.D.), i.e., mean +2*S.D. of the degree distribution, were taken as hubs, i.e., High Connectivity (HC) nodes [21]. (**Table 3**)
2. In the second step Betweenness centrality was taken as parameter to extract significant nodes. Betweenness centrality of the nodes in the QQPPI networks (**Figure 2** **,** **3** **, S1**) showed a varied distribution. Only a handful of nodes had betweenness score greater than 1000. However, almost 40–45% of nodes had zero betweenness. The node betweenness distribution was sorted in descending order and nodes with betweenness score lying in the top 50% of the distribution were selected. Among these sorted nodes, the nodes identified with degree less than the cut-off degree for HC nodes and directly connected to at least 2 HC nodes were selected as bottlenecks, i.e., High Betweenness but Low Connectivity (HBLC) nodes.

**Table 3:** Cut-off determination for hubs (HC nodes).

GeneChip A (HG_133A)			
	Mean (M)	Standard Deviation (S)	Cut-off (M+2*S)
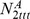	3.4	4.2	11.8≈12
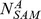	2.8	2.9	8.6≈9

### Identification of cliques

In this study, cliques with 3 nodes and 4 nodes (3-clique, 4-clique) were identified in 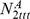, 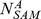 and 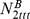. The cliques were identified with the help of a self developed algorithm (**File S1**). To validate the authenticity and correctness of the algorithm, it was simulated for the network obtained from POINeT and the output of the program was compared with the list of cliques given in POINeT for that network, the results exactly matched. The development of the in house algorithm was necessary to find the cliques (three and more) in the merged networks (obtained from POINeT and Genes2FANs). Only 3-Cliques and 4-Cliques were obtained, and higher order cliques were absent in the network.

### Identification of complexes containing clique forming proteins

A protein complex is a complex containing multiple proteins that interact with each other. They are in the form of quaternary structure, and the proteins in the complex are linked by non-covalent protein-protein interactions. The complexes in the PPI networks were identified with the help of the database CORUM [22]. The clique forming proteins were given as query in the CORUM database to find out the complexes containing this proteins. Furthermore, with the help of an in house algorithm (**File S2**) all the proteins associated with a specific complex were identified. A cut-off for the number of query proteins in a complex is assigned. For 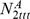, comlexes containing 5 or more query proteins were listed. Similarly for 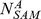, complexes containing 4 or more query proteins were listed. In 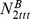, since only 2 proteins are involved in a particular complex, we did not consider this QQPPI network for complex detection. The programs to find cliques and complex have been implemented using C language, compiled and tested on Windows 7 Professional edition.

**File S3** lists the plots of connectivity distribution and betweenness distribution of the three QQPPI networks (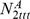, 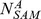, 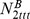).

### Gene level co-expression analysis of interacting proteins

Pearson correlation coefficient was used to find out the gene level co-expression of interacting proteins in the QQPPI networks (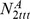, 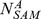 and 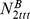). In the QQPPI networks, gene level co-expression of each pair of interacting proteins was used to assign weight to the edges of the network. Percentage change in co-expression of interacting proteins was also calculated.

### Comparison with the study of Moran *et al.* [10]

Different analytic approaches can be taken to analyse the same microarray data with different set of goals [7]. The original contributors of the microarray data series GSE8397 were Moran *et al.* who focused on establishing the transcriptomic expression profile of the medial & lateral substantia nigra and the superior frontal cortex. The differentially regulated genes identified in their study were compared to the results of our study.

## Results & Discussion

### Study of Differential Expression (DE) of genes

Involvement of substantia nigra (SN) in PD is well known [23], [24], [25]. PD related motor symptoms mainly occur due to the depletion of up to 60% of dopaminergic neurons and aggregation of round, hyaline neuronal cytoplasmic inclusions called Lewy Bodies (LBs) in SN [24], [25]. Significant involvement of frontal cortex in PD has also been reported [10], [25], [26]. The dataset (GSE8397) provided by Moran *et al.* [10] is the only available dataset till date which covers the tissue samples both from substantia nigra and frontal cerebral cortex. Therefore we have considered these datasets for our study.

Initially the microarrays in GSE8397 were analyzed using 2-tailed *t*-test. Each disease sample group was paired with the control sample group in the *t*-tests. 2-tailed *t*-test is a measure of the statistical significance of the dataset, in terms of a test statistic *t*, which is given by:
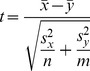
(1)where 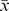 and 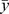 are the sample means, 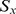 and 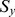 are the sample standard deviations, *n* and *m* are the sample sizes for two samples, *x* and *y*. Under the null hypothesis, this test returns the probability (P value) of observing a value as extreme or more extreme of the test statistic. Probes corresponding to a portion of the genes showed significant changes in signal intensities in disease sample groups, as compared to the control. These genes were selected as Differentially Expressed (DE) genes.

Previously, 2-tailed *t*-test has been successfully used to select differentially expressed data from microarray datasets [7]. However, 2-tailed *t*-test does not give any up-regulated or down-regulated gene information. Therefore, Significance Analysis of Microarrays (SAM) was used to identify up-regulated (UR) or down-regulated (DR) DE genes in the disease state. SAM calculates a test statistic for relative difference in gene expression based on permutation analysis of expression data, and False Discovery Rate [27] which is given by:
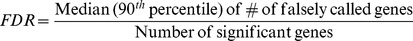
(2)


In SAM, Fold changes are also specified to guarantee that significant genes change at least at a pre-specified amount. This means that the absolute value of the average expression levels of a gene under each of two conditions must be greater than the fold change to be called positive and less than the inverse of the fold change to be called negative. This way, SAM gives better result in terms of differential expression than 2-tailed *t*-test as the latter does not take into account fold changes to determine significance of average gene expression levels.

1443 and 1518 DE genes were reported using 2-tailed *t*-test (P values<0.001) and SAM (FDR 0.19%) respectively from GeneChip A (HG_U133A). Out of the 1518 SAM reported DE genes, 293 genes were up-regulated (UR) and 1225 were down-regulated (DR).

Similar methodology (2-tailed *t*-test at P values<0.001 and SAM at FDR 0.19%) was followed to analyse GeneChip B (HG_U133B), but no significant DE gene was found. However when we increased the P value (P<0.05) of 2-tailed *t*-test, 1606 genes were found to be DE.

These DE genes were selected for subsequent ontological analyses followed by network analyses as their abnormal gene expression profiles in disease state indicated probable involvement in disease pathology.

### Functional analysis of DE genes

The DE genes were subjected to FatiGO [14] for functional analysis. The over-representative GO terms (P value<0.05) were considered. Among these GO terms, the over-representative biological processes showed large number of DE genes as compared to other GO terms and KEGG pathways (**Table 1**). Therefore, the DE genes involved in the biological processes were selected in our study for subsequent network generation based on a similar approach presented in a previous study [28]. For the dataset obtained from GeneChip A (HG_U133A) using 2-tailed *t*-test (P<0.001), 779 genes (distributed among 792 biological processes) were chosen as significant DE genes (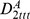). Similarly, for the dataset obtained from GeneChip A (HG_U133A) using SAM, 207 genes (distributed among 381 biological processes) were chosen as significant DE genes (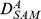). For the dataset obtained from GeneChip B (HG_U133B) using 2-tailed *t*-test (P<0.05), 221 genes (distributed among 61 biological processes) were chosen as the significant DE genes (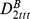).

### Topological analyses of QQPPI networks

A PPI network is commonly represented as an undirected (edges have no direction) graph, 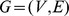, where 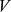 is the set of nodes (proteins) and 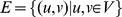 is the set of edges (protein interactions). Thus the networks we studied are undirected and unweighted protein-protein interaction networks based on DE genes of PD microarray data.

QQPPI networks can be characterized by several topological parameters. Out of these, one of the most basic yet essential parameter is node degree, or connectivity. It signifies the number of edges incident on particular node. For a node 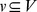, the set of edges incident on 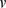 is denoted as 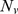, where 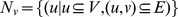. The cardinality of 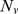, i.e., 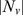 is 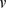
's connectivity, or degree in *G*, also known as 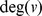. High connectivity (HC) of a node indicates that the node (protein) has direct interaction (physical interaction and/or complex formation) with many other distinct nodes (proteins). Proteins with high connectivity are considered to be essential hubs of the network, whose removal would result in an overall collapse of the global structure of the network [6]. We have extracted hubs from the QQPPI networks using the criterion described in section 2.4. **Table 4** gives the number of hubs obtained from the QQPPI networks. Hub genes identified in the QQPPI networks are listed in **Table 5** **,** **6** and **7**. Betweenness centrality of a node 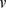 is given by the expression:
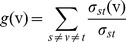
(3)where 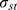 is the total number of shortest paths from node *s* to node *t*, and 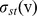 is the total number of shortest paths that pass through 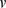. Betweenness centrality quantifies the flow of information through a node in the network. In case of a PPI network, it specifies how a node influences the communication among other nodes. Therefore, in a QQPPI network, betweenness centrality helps to locate important but not very highly connected nodes.

**Table 4:** Number of obtained hubs (HC nodes) & bottlenecks (HBLC nodes).

GeneChip A (HG_133A)		
	Number of hubs (HC nodes)	Number of bottlenecks (HBLC nodes)
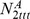	19	41
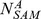	5	14

**Table 5:** Hubs & bottlenecks in 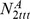.

**Hubs**	YWHAZ, ACTB, ACTG1, YWHAB, YWHAE, MAPK1, MAP3K14, DISC1, APP, VCL, VIM, FTSJ1, HSPA1A, IKBKB, YWHAQ, ARRB2, EEF1A1, SNCA, SQSTM1
**Bottlenecks**	HDAC4, TGFB1, STUB1, ANXA2, KPNB1, SET, STX1A, SPTBN1, AXIN1, IQGAP1, RAD23A, RPS3, CHUK, MCL1, DAPK1, PARD3, TJP2, ACTN2, TAF9, IGF1R, CDC25B, IARS, CTNNA1, PTPN3, IRAK1, TFRC, VASP, MAP3K7IP2, ADAM17, CYCS, MAP2K4, WEE1, SF3B1, DSTN, SRRM2, BAG2, C1QBP, PHB, YWHAH, GSN, MARCKS

**Table 6:** Hubs & bottlenecks in 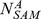.

**Hubs**	YWHAZ, YWHAB, CSNK2A1, CLTC, CDC42
**Bottlenecks**	PRKCZ, APC, SNCA, NFKBIA, IQGAP1, TSC2, IGF1R, HSPA1A, OCRL, PARD3, CLTB, TH, ATP5A1, TUBB

**Table 7:** Hubs & bottlenecks in 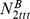.

**Hubs**	MAPK1, YWHAG, MAPK8, ACTB, PAK1
**Bottlenecks**	CDC42, MAP3K2, MAP1B, MBP, NDE1, DUSP1, AKT2

Current studies [29]–[32] have shown that node connectivity might not be the only influential parameter to characterize biological networks. Goñi *et al.* [33] described that in case of neurodegenerative diseases, less extensively connected proteins are much more appropriate therapeutic targets than highly connected ones, as the critical role of highly connected nodes (hubs) in the network modules prevent them from substantial fluctuation. Recently, it was shown that betweenness centrality can also be an important parameter for finding lowly connected (non-hub) but important nodes [34], [35].

**Figure 4:**
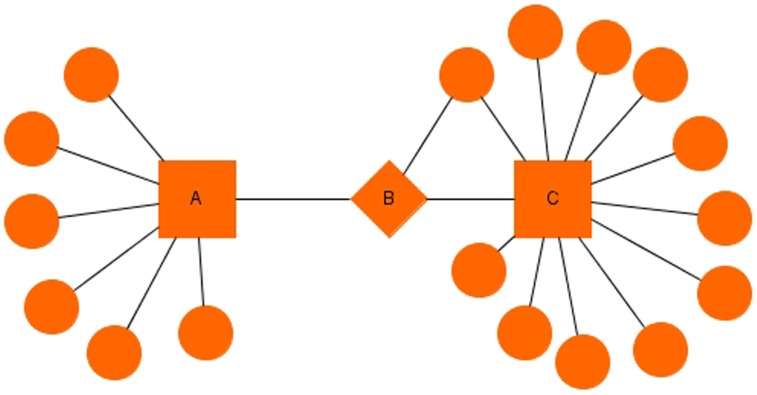
Graphical structure of a simple PPI network. High Connectivity (HC) nodes or hubs: A & C. High Betweenness but Low Connectivity (HBLC) nodes or bottlenecks: B.

Proteins with low connectivity but high betweenness may play a key role in the modular structure in the yeast interactome. Gursoy *et al.* [36] studied the properties of High Betweenness but Low Connectivity (HBLC) nodes, and their importance in the context of biological networks. The Highly betweened but lowly connected nodes are also considered as bottlenecks [35]. Yu *et al.* [35] Suggested that HBLC nodes are more essential, and betweenness is found to be a more significant indicator of essentiality than degree. **Table 4** gives the number of bottlenecks obtained from the QQPPI networks. **Table 5** **,** **6** and **7** gives the bottlenecks of our QQPPI networks. **Figure 4** represents the graphical structure of a simple PPI network containing hubs and bottlenecks. **Table S1**, **S2** and **S3** lists all the nodes, hubs and bottlenecks in 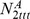, 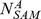 and 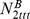 along with their topological parameters as obtained from tYNA.

### Identification of cliques & complexes

A clique 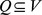 is a subset of the vertices of 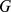 (refer to section 3.3) such that 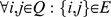. In a PPI network, a clique signifies that every pair of proteins physically interacts with each other. Cliques have been used to identify functional units [37] and physical complexes [38] in PPI networks. Several three and four cliques were identified in the QQPPI networks using a self-developed algorithm (refer to section 2.5). Most of these cliques are overlapping. **Table 8** shows the number of cliques identified in the QQPPI networks (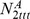, 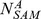 and 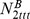). **Table 9** shows the complexes formed by individual and overlapping cliques in 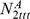 and 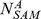.

**Table 8:** Numbers of 3 and 4-cliques in the QQPPI networks.

GeneChip A (HG_133A)		
	3-cliques	4-cliques
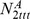	126	12
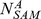	29	2

**Table 9:** List of complexes for the network 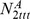 and 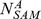.

Gene names	Complex*
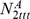	
CD2BP2, DDX17, NF2, PRPF8, SF3A2, SF3B1, SRRM2, WBP11	Spliceosome (ID: 351)
ACTB, ACTG1, NF2, SMARCA4, SMARCC1, SMARCC2	Polybromo and BAF containing complex(ID: 149, 189)
MAP2K1, MAPK1, YWHAB, YWHAE, YWHAH, YWHAZ	Ksr1 complex (ID: 5909, 5937)
ACTB, NF2, SMARCA4, SMARCC1, SMARCC2	Nucleosomal methylation activator complex (ID: 86), BAF complex(ID: 566), LARC complex (ID: 778)
ACTB, NF2, SMARCC1, SMARCC2, VDR	Emerin complex 32(ID: 5614)
APC, APP, PSMC4, PSMD1, PSMD4	Proteasome (ID: 181, 193)
EEF1A1, MYB, RPLP0, RPLP1, RPS3	Nop56p-associated pre-rRNA complex(ID: 3055)
MAP2K1, YWHAB, YWHAE, YWHAH, YWHAZ	Ksr1 complex(ID: 5886, 5936)
NF2, SMARCA4, SMARCC1, SMARCC2, VDR	WINAC complex(ID: 1230)
PNN, PRPF8, SF3A2, SF3B1, SRRM2	C complex spliceosome (ID: 1181)
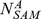	
AMPH, AP2A2, AP2M1, EPS15, TH	Epsin-clathrin complex (ID: 1228)
AMPH, CLTC, DNM1, EPS15, SYNJ1	Endocytic coat complex (ID: 5344)
AMPH, CLTC, DNM1, SYNJ1, TH	Endocytic coat complex (ID: 5345)
APC, PSMA1, PSMB2, PSMB7, TH	Proteasome complex(ID: 181, 191, 192, 193, 194)
APC, CDC42, IQGAP1, TH	APC-IQGAP1-Rac1 complex (ID: 3011), APC-IQGAP1-Cdc42 complex (ID: 3012)
CDC42, PARD3, PRKCZ, TH	CDC42-Par6c-Par3-Prkcz complex (ID: 804) Tiam1-Par-3-aPKC-zeta complex (ID: 1023)
CSNK2A1, TH, YWHAB, YWHAZ	Ksr1-CK2-MEK-14-3-3 complex, PDGF treated (ID: 5936)

* The complexes are given along with their CORUM IDs.

For each QQPPI network (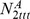, 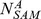 and 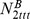), 3-cliques and 4-cliques were combined to detect tightly knitted sub-networks, which are the core functional modules in the QQPPI networks [7] (**Figure 2** **,** **3** **, S1**). **Table S4** lists the nodes in the functional modules, along with their connectivity, betweenness, and their numbers of occurrences in 3- and 4-cliques. For each QQPPI network, it can be observed that most of the hubs and bottlenecks belonged to the core functional modules. Several cliques in the sub-networks belonging to 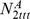 and 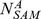 were found to be involved in already known protein complexes (**Table 9**).

### Gene level co-expression analysis of proteins interacting within a complex

The Pearson correlation coefficient (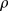) is a measure of the linear dependence between two variables giving a value between +1 and −1 inclusive. It is used as a measure of the strength of linear dependence between two variables. It is defined as the covariance of the two variables divided by the product of their standard deviations.

**Table 10** and **Table 11** lists the values of Pearson correlation coefficient (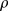) of two interacting complex forming nodes and their change in both control and disease states (in 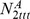 and 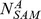 respectively). **Table S5**, **S6** and **S7** shows the Pearson correlation coefficient (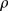) of proteins interacting within cliques, along with net difference of 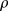 between control and disease samples and their percentage of maximum possible change, in the core functional modules detected in 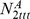, 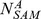 and 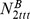 respectively.

**Table 10:** Co-expression analysis of proteins interacting within a complex (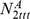).

Node 1	Node 2	Control (C)	Disease (D)	Change (C–D)	Complex
CD2BP2	PRPF8	0.182452	0.377959	−0.195506	Spliceosome (ID: 351)
CD2BP2	SF3A2	−0.49456	0.300699	−0.795258	
CD2BP2	WBP11	0.064197	−0.18898	0.253176	
PRPF8	SF3A2	−0.09299	−0.01542	−0.07757	
SF3A2	SF3B1	0.236131	−0.15777	0.393901	
SF3A2	SRRM2	0.210102	0.531593	−0.321490	
SF3A2	WBP11	−0.10021	−0.16228	0.062070	
ACTB	ACTG1	0.344993	0.571264	−0.226271	Polybromo and BAF containing complex(ID: 149, 189)
ACTB	SMARCA4	0.272319	0.085326	0.186992	
ACTG1	NF2	−0.2271	0.0839	−0.311	
SMARCA4	SMARCC1	−0.32228	−0.2122	−0.110080	
SMARCC1	SMARCC2	0.197918	0.250794	−0.052876	
MAP2K1	MAPK1	0.705468	0.59637	0.109098	Ksr1 complex (ID: 5909, 5937)
YWHAE	YWHAH	−0.30185	−0.16978	−0.132070	
YWHAE	YWHAZ	−0.24657	−0.06473	−0.18184	
PSMC4	PSMD1	0.305936	0.155939	0.149996	Proteasome (ID: 181, 193)
PSMC4	PSMD4	0.030223	0.49093	−0.460706	
PSMD1	PSMD4	0.129277	0.429546	−0.300269	
EEF1A1	RPLP1	−0.62308	−0.37849	−0.244589	Nop56p-associated pre-rRNA complex(ID: 3055)
PRPF8	SF3A2	−0.09299	−0.01542	−0.07757	C complex spliceosome (ID: 1181)
SF3A2	SF3B1	0.236131	−0.15777	0.393901	
SF3A2	SRRM2	0.210102	0.531593	−0.321490	

**Table 11:** Co-expression analysis of proteins interacting within a complex (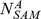).

Node 1	Node 2	Control (C)	Disease (D)	Change (C–D)	Complex
AMPH	AP2A2	0.895323	0.711618	0.183705	Epsin-clathrin complex (ID: 1228)
AP2A2	AP2M1	0.896811	0.71299	0.183821	
AP2A2	EPS15	0.680562	0.130673	0.549888	
AMPH	CLTC	0.835258	0.766836	0.068421	Endocytic coat complex (ID: 5344)
AMPH	DNM1	0.859907	0.771107	0.088799	
CLTC	EPS15	0.594193	0.346006	0.248186	
CLTC	SYNJ1	0.645271	0.769517	−0.124245	
DNM1	EPS15	0.752444	0.283483	0.468961	
EPS15	SYNJ1	0.694805	0.449957	0.244848	
PSMA1	PSMB2	0.389236	0.512285	−0.123048	Proteasome complex(ID: 181, 191, 192, 193, 194)
PSMA1	PSMB7	0.515788	0.620496	−0.104708	
PSMB2	PSMB7	0.102045	0.310781	−0.208735	
APC	IQGAP1	−0.61332	−0.33299	−0.280329	APC-IQGAP1-Rac1 complex (ID: 3011), APC-IQGAP1-Cdc42 complex (ID: 3012)
CDC42	IQGAP1	−0.18484	−0.2834	0.098559	
CDC42	PARD3	−0.13129	−0.35908	0.227790	CDC42-Par6c-Par3-Prkcz complex (ID: 804), Tiam1-Par-3-aPKC-zeta complex (ID: 1023)
CDC42	PRKCZ	0.762057	0.706097	0.055960	
PARD3	PRKCZ	−0.13645	−0.44763	0.311180	
CSNK2A1	YWHAB	0.253246	0.672328	−0.419082	Ksr1-CK2-MEK-14-3-3 complex, PDGF treated (ID: 5936)
TH	YWHAB	−0.47327	0.157579	−0.630848	
TH	YWHAZ	−0.36101	−0.1174	−0.243609	

Spliceosome complex (ID: 351) has been found to be the most significant in terms of change in co-expression in 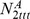 (**Table 10**). Moreover, Ksr1-CK2-MEK-14-3-3 complex, PDGF treated (ID: 5936) shows significant difference in co-expression value in 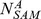 (**Table 11**).

### Association of disease markers with cliques and neurotransmitters

Having identified the topologically significant (HC and HBLC) nodes, we then set out to study their association with PD. We used Genotator meta-database [39] and the text mining engine PubMed (http://www.ncbi.nlm.nih.gov/pubmed) for this purpose. 13 hubs and 15 bottlenecks in 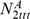 and 3 hubs and 9 bottlenecks in 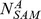 were found to be associated with PD (**Table 12**). However, 6 hubs, 26 bottlenecks in 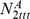 and 2 hubs, 5 bottlenecks in 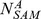 were unreported for PD (**Table 13** **,** **14**). Due to the lack of topologically significant nodes in, 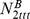 we did not consider 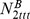 for further analysis. Thus 39 (6+26+2+5 = 39) nodes were obtained from our QQPPI networks which were not previously known to be associated with PD. Among these 39 nodes, 2 nodes (IQGAP1 and PARD3) were common for both 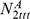 and 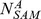. Therefore, these 37 (39−2 = 37) topologically significant nodes (hubs & bottlenecks) were considered as disease biomarkers in our study. The list of these genes, along with their symbols, names and brief description of their functions are shown in **Tables 15** and **16**.

**Table 12:** Previously reported PD-associated disease markers in 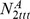 and 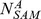.

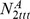	
**Hubs**	YWHAZ, YWHAB, YWHAE, MAPK1, DISC1, APP, VCL, VIM, HSPA1A, IKBKB, YWHAQ, SNCA, SQSTM1
**Bottlenecks**	HDAC4, TGFB1, SET, SPTBN1, RAD23A, RPS3, CHUK, DAPK1, IGF1R, IRAK1, CYCS, MAP2K4, SRRM2, PHB, YWHAH
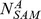	
**Hubs**	YWHAZ, YWHAB, CDC42
**Bottlenecks**	PRKCZ, APC, SNCA, NFKBIA, TSC2, IGF1R, HSPA1A, TH, ATP5A1

**Table 13:** Previously unreported disease markers in 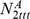.

Hubs	Degree	Number of occurrence in 3-cliques	Number of occurrence in 4-cliques
* **ACTB**	29	17	6
* **ACTG1**	23	22	5
MAP3K14	18	14	5
FTSJ1	13	1	0
# **ARRB2**	12	8	2
EEF1A1	12	5	1

Bottlenecks	Betweenness	Number of occurrence in 3-cliques	Number of occurrence in 4-cliques
STUB1	2669.638323	4	1
ANXA2	2425.615147	4	1
KPNB1	2313.064649	4	0
# **STX1A**	1758.534123	1	0
AXIN1	1649.015233	0	0
IQGAP1	1591.554704	5	0
MCL1	1149.028538	0	0
PARD3	1031.756667	0	0
TJP2	995.5834285	4	1
ACTN2	989.1836942	2	0
TAF9	973.4038033	0	0
CDC25B	876.2708288	3	0
IARS	773.0306479	1	0
* **CTNNA1**	695.7911325	8	2
PTPN3	666.0363488	0	0
# **TFRC**	522.4965384	1	0
VASP	500.2301461	3	1
MAP3K7IP2	484.3384659	1	0
ADAM17	464.6173296	0	0
WEE1	392.7818697	0	0
SF3B1	375.7263891	2	0
DSTN	342.8748485	1	0
BAG2	253.0975395	4	1
C1QBP	236.1694422	2	0
* **GSN**	110.0727932	6	2
# **MARCKS**	98.75040177	0	0

*Topologically significant disease markers.

# disease markers associated with dopamine and other neurotransmitters.

**Table 14:** Previously unreported disease markers in 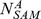.

Hubs	Degree	Number of occurrence in 3-cliques	Number of occurrence in 4-cliques
* **CSNK2A1**	12	3	0
* **CLTC**	11	7	1

Bottlenecks	Betweenness	Number of occurrence in 3-cliques	Number of occurrence in 4-cliques
* **IQGAP1**	284.1779164	2	0
OCRL	97.31967486	0	0
* **PARD3**	79.45372874	3	0
CLTB	77.62499748	0	0
TUBB	23.27462677	0	0

*Topologically significant disease markers.

**Table 15:** Brief description of previously unreported disease markers in 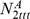.

Hubs (Official symbol)	Full name	Brief description
*ACTB	actin, beta	This gene encodes one of six different highly conserved actin proteins. which are involved in cell motility, structure, and integrity.
*ACTG1	actin, gamma 1	Actins are highly conserved proteins that are involved in various types of cell motility, and maintenance of the cytoskeleton.
MAP3K14	mitogen-activated protein kinase kinase kinase 14	It is a serine/threonine protein-kinase which binds to TRAF2 and stimulates NF-kappaB activity.
FTSJ1	FtsJ RNA methyltransferase homolog 1 (E. coli)	It encodes a member of the methyltransferase superfamily which localizes to the nucleolus, binds to S-adenosylmethionine, and may be involved in the processing and modification of ribosomal RNA.
#ARRB2	arrestin, beta 2	This protein exhibits sensitized dopamine release in mice.
EEF1A1	eukaryotic translation elongation factor 1 alpha 1	This gene encodes an isoform of the alpha subunit of the elongation factor-1 complex, which is responsible for the enzymatic delivery of aminoacyl tRNAs to the ribosome.
Bottlenecks (Official symbol)		
STUB1	STIP1 homology and U-box containing protein 1, E3 ubiquitin protein ligase	It is a ubiquitin ligase/cochaperone that participates in protein quality control by targeting a broad range of chaperone protein substrates for degradation.
ANXA2	annexin A2	This gene encodes a member of the calcium-dependent phospholipid-binding protein family and plays a role in the regulation of cellular growth and in signal transduction pathways.
KPNB1	karyopherin (importin) beta 1	The protein encoded by this gene is a member of the importin beta family which interacts with the FG repeats of nucleoporins for translocation through the pore complex.
#STX1A	syntaxin 1A (brain)	Syntaxin 1A regulates dopamine transporter activity, phosphorylation and surface expression.
AXIN1	axin 1	This gene encodes a cytoplasmic protein which contains a regulation of G-protein signaling (RGS) domain and a dishevelled and axin (DIX) domain.
MCL1	myeloid cell leukemia sequence 1 (BCL2-related)	This gene encodes an anti-apoptotic protein, which is a member of the Bcl-2 family.
TJP2	tight junction protein 2	This gene encodes a zonula occluden that is a member of the membrane-associated guanylate kinase homolog family which functions as a component of the tight junction barrier in epithelial and endothelial cells.
ACTN2	actinin, alpha 2	Alpha actinins belong to the spectrin gene superfamily which represents a diverse group of cytoskeletal proteins, including the alpha and beta spectrins and dystrophins.
TAF9	TAF9 RNA polymerase II, TATA box binding protein (TBP)-associated factor, 32 kDa	Protein encoded by this gene participates in basal transcription, serve as coactivators, function in promoter recognition or modify general transcription factors (GTFs) to facilitate complex assembly and transcription initiation.
CDC25B	cell division cycle 25B	CDC25B is a member of the CDC25 family of phosphatases which activates the cyclin dependent kinase CDC2 by removing two phosphate groups and it is required for entry into mitosis.
IARS	isoleucyl-tRNA synthetase	It catalyzes the aminoacylation of tRNA by their cognate amino acid. It is thought to be among the first proteins that appeared in evolution.
*CTNNA1	catenin (cadherin-associated protein), alpha 1, 102 kDa	Protein encoded by this gene associates with the cytoplasmic domain of a variety of cadherins.
PTPN3	protein tyrosine phosphatase, non-receptor type 3	The protein encoded by this gene is a member of the protein tyrosine phosphatase (PTP) family which are signaling molecules that regulate a variety of cellular processes including cell growth, differentiation, mitotic cycle, and oncogenic transformation.
#TFRC	transferrin receptor	It is necessary for development of erythrocytes and the nervous system.
VASP	vasodilator-stimulated phosphoprotein	It is a member of the Ena-VASP protein family. It contains an EHV1 N-terminal domain that binds proteins containing E/DFPPPPXD/E motifs and targets Ena-VASP proteins to focal adhesions.
MAP3K7IP2	MAP3K7 binding protein 2	The protein encoded by this gene is an activator of MAP3K7/TAK1, which is required for for the IL-1 induced activation of nuclear factor kappaB and MAPK8/JNK.
ADAM17	ADAM metallopeptidase domain 17	This gene encodes a member of the ADAM (a disintegrin and metalloprotease domain) family which has been implicated in a variety of biologic processes like fertilization, muscle development, and neurogenesis.
WEE1	WEE1 homolog (S. pombe)	This gene encodes a nuclear protein, which is a tyrosine kinase belonging to the Ser/Thr family of protein kinases.
SF3B1	splicing factor 3b, subunit 1, 155 kDa	This gene encodes subunit 1 of the splicing factor 3b protein complex.
DSTN	destrin (actin depolymerizing factor)	The product of this gene belongs to the actin-binding proteins ADF family which is responsible for enhancing the turnover rate of actin in vivo.
BAG2	BCL2-associated athanogene 2	BAG proteins compete with Hip for binding to the Hsc70/Hsp70 ATPase domain and promote substrate release.
C1QBP	complement component 1, q subcomponent binding protein	It associates with C1r and C1s in order to yield the first component of the serum complement system and is known to bind to the globular heads of C1q molecules and inhibit C1 activation.
*GSN	Gelsolin	The protein encoded by this gene binds to the “plus” ends of actin monomers and filaments to prevent monomer exchange.
#MARCKS	myristoylated alanine-rich protein kinase C substrate	The protein encoded by this gene is a substrate for protein kinase C. It is localized to the plasma membrane and is an actin filament crosslinking protein.

*Topologically significant disease markers.

# disease markers associated with dopamine.

**Table 16:** Brief description of previously unreported disease markers in 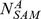.

Hubs (Official symbol)	Full name	Brief description
*CSNK2A1	casein kinase 2, alpha 1 polypeptide	It phosphorylates acidic proteins such as casein.
*CLTC	clathrin, heavy chain	It is a major protein component of the cytoplasmic face of coated vesicles and coated pits, which is involved in the intracellular trafficking of receptors and endocytosis of a variety of macromolecules.
Bottlenecks (Official symbol)		
*IQGAP1	IQ motif containing GTPase activating protein 1	This gene encodes a member of the IQGAP family and interacts with components of the cytoskeleton, with cell adhesion molecules, and with several signalling molecules to regulate cell morphology and motility.
OCRL	oculocerebrorenal syndrome of Lowe	This gene encodes a phosphatase enzyme that is involved in actin polymerization and is found in the trans-Golgi network.
*PARD3	par-3 family cell polarity regulator	This gene encodes a member of the PARD protein family which affects asymmetrical cell division and direct polarized cell growth.
CLTB	clathrin, light chain B	Clathrin is a large, soluble protein composed of heavy and light chains which functions as the main structural component of the lattice-type cytoplasmic face of coated pits and vesicles.
TUBB	tubulin, beta class I	It is the major constituent of microtubules which binds two moles of GTP, one at an exchangeable site on the beta chain and one at a non-exchangeable site on the alpha chain.

*Topologically significant disease markers.

These 37 unique disease markers (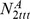 and 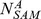) were then subjected to detailed analysis about their association in cliques and neurotransmitters. Interestingly it was found that 8 (CSNK2A1, CLTC, PARD3, IQGAP1, ACTB, ACTG1, CTNNA1 and GSN) out of the 37 nodes were strongly associated with cliques that form the core functional modules of the networks. Furthermore, significant changes in co-expression levels were observed between control and disease states in most of these core forming nodes (**Table 17**).

**Table 17:** Co-expression level of significant disease markers in core functional modules.

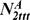 -contained core functional module			
Hubs	Interacting partners	Control*	Disease*
ACTB	TJP2	0	−0.33
	VCL	−0.2	0.13
	RPLP0	−0.21	0.18
	ANXA2	−0.47	0.03
	ARPC1B	−0.38	0.03
	HSPA1B	0.43	−0.28
ACTG1	NF2	−0.12	0.03
	MAP3K7IP2	−0.31	0
	VCL	−0.27	0.2
	IQGAP1	0	−0.2
	VASP	0.03	−0.16
	SPTBN1	0.24	−0.19
	TJP2	−0.15	0.05
	ARPC1B	−0.33	0.14

Bottlenecks	Interacting partners	Control*	Disease*
CTNNA1	VCL	0.14	−0.06
	SPTBN1	−0.2	0.03
GSN	ACTB	0.16	−0.23
	VASP	0.41	−0.53

Bottlenecks	Interacting partners	Control*	Disease*
PARD3	*No significant co-expression change available*		
IQGAP1	YWHAZ	−0.39	0.25

*Here the co-expression values are rounded up to the second decimal place.

PD is characterised by the loss of dopaminergic neurons in the subsantia nigra pars compacta [40]. Association of PD and loss of dopamine neurotransmitter has been established [24]. Other than dopamine, several neurotransmitters viz., choline, serotonin, noradrenaline, glutamate and GABA are also involved with PD-specific motor and non-motor symptoms [23]. We studied the association of the 37 unreported genes with any of these neurotransmitters. Four (ARRB2, STX1A, TFRC and MARCKS) out of the 37 markers were found to be associated with several neurotransmitters including dopamine (**Table 18**) [40]–[52].

**Table 18:** Involvement of unreported disease markers (in 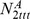) with neurotransmitters.

	Dopaminergic	Cholinergic	Serotonergic	Adrenergic	Glutamatergic	GABAergic
ARRB2	**+** [40], [41]	**−**	**−**	**+** [42]	**−**	**−**
STX1A	**+** [43]	**−**	**+** [44], [45], [46]	**−**	**+** [47]	**+** [47]
TFRC	**+** [48]	**−**	**−**	**−**	**−**	**−**
MARCKS	**+** [49]	**−**	**+** [50]	**+** [50], [51]	**+** [52]	**−**

+ indicates association. − indicates no association. Corresponding references for association are shown within third brackets.

These 37 unreported proteins may be considered as important disease marker genes. However, the 8 clique-forming proteins and the 4 neurotransmitter (including dopamine) associated proteins showed significant topological and functional importance in the QQPPI networks. Therefore, these 12 (8+4) proteins may be considered as key disease markers or biomarkers for PD. These proteins are called biomarkers due to five different reasons (1) These were found to be differentially expressed in PD-related microarray datasets (2) Proteins corresponding to these genes are the most topologically significant nodes (hubs and bottlenecks) in the protein-protein interaction networks (3) They showed significant involvement in the known complexes (4) They showed involvement with PD-associated neurotransmitters (5) These were not known previously to be associated with PD.

### Comparison with the study of Moran *et al.*

Moran *et al.* reported several genes to be confirmed PD-associated sequences or a first PD expression signature [10]. A very important finding of this study concerned a series of 25 highly DE sequences which map to known PARK loci. It was proposed in their study that these 25 sequences represented candidates for as yet unidentified disease-causing genes. Interestingly, results of our study had very little overlap with their outcomes. Out of the 25 sequences reported in their study, only 1 was common to the data points in 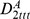 (VAV3), 3 were common to the data points in 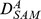 (MDH1, VAV3, CDC42) and 1 was common to the data points in 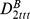 (CDC42). Out of these, CDC42 was the only protein which acted as a significant node: as a hub in 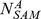 and as a bottleneck in 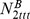. Here it is interesting to note that CDC42 was recently proposed in a PPI network-based study to play critical roles in PD [53].

However, one should keep in mind that these studies had different goals. Hence the difference in the final outcomes is quite obvious. Also, this study takes into account an extensive statistical, topological and functional analysis to determine significant disease markers which was not performed in the previous study

## Limitations

Genes2FANs combines protein interaction data from DIP [54], MINT [55], BIND [56], HPRD [57], BioGRID [58], InnateDB [59], KEGG [60], IntAct [61], PPID [62], Ma'ayan *et al.* [63], Stelzl *et al.* [64], Rual *et al.* [65] and Yu *et al.* [66]. Similarly, POINeT combines protein interaction data from DIP, MINT, BIND, HPRD, BioGRID, IntAct, MIPS [67], CYGD [68] and MPact [69]. Hence, by the merger of QQPPI networks formed by both Genes2FANs and POINeT, it was possible to access PPI data from all of these 14 databases in this study. Any insufficient and non-updated information in the databases will have an effect on our results. To minimize this error, we performed our studies using the information of the above mentioned databases updated till May, 2014. However information in most of the databases is incomplete. Hence, markers whose PPI data were not included in the databases in the above mentioned open source databases could not be included in this study.

Furthermore, the incompleteness of the human interactome could lead to data insufficiency, resulting in biased topological analyses. In this study, the PPI networks were constructed based on the assumption that the expression level of most of the proteins and mRNAs were positively correlated, but this might not be true for all cases. Furthermore, due to post-transcriptional and translational regulations, the correspondence between expression of a gene and its protein is complicated. It was not possible to incorporate protein expression in our study.

## Conclusion

Differentially expressed genes in post-mortem brain samples of patients with PD have been identified in this study. Gene expression data and PPI data were used for topological analyses of protein-protein interactions for PD. Two sets of DE genes were selected from the microarray data separately using 2-tailed *t*-tests and SAM. These two sets of DE genes were run separately to construct QQPPI networks. Several important topologically significant nodes e.g., hubs and bottlenecks were identified as biologically significant nodes in the network, as it has already been established that hubs and bottlenecks correspond to biologically significant proteins with respect to the disease. With this approach, we have identified 37 proteins in our QQPPI networks which were not previously known to be associated with PD. Three and four-cliques were identified in the QQPPI networks. These cliques contain most of the topologically significant nodes of the networks which form core functional modules consisting of tightly-knitted sub-networks. Several cliques identified in our study were found to be involved in already known protein complexes associated with many biological processes. Out of the 37 markers, eight (CSNK2A1, CLTC, PARD3, IQGAP1, ACTB, ACTG1, CTNNA1 and GSN) were significantly involved in the core functional modules and showed significant change in co-expression levels between disease and control state. Furthermore, proteins encoded by 4 genes (ARRB2, STX1A, TFRC, MARCKS) showed involvement with several neurotransmitters including dopamine, which plays a significant role in PD. These 12 proteins may be considered as biologically significant with respect to PD. Our study represents a novel investigation of the PPI networks for PD. The 37 network biomarkers identified in our study may provide as potential therapeutic targets for PD applications developments.

## Supporting Information

Figure S1 **QQPPI network built from the dataset obtained using 2-tailed** ***t*** **-test (P<0.05) (GeneChip B).** Orange coloured square nodes represent hubs (HC nodes). Yellow coloured triangular nodes represent bottlenecks (bottlenecks). The core functional module containing 3,4-cliques are represented using blue coloured edges. Non-hub non-bottleneck nodes are coloured green if they are directly connected to a hub or a bottleneck, and grey otherwise. Inset: Subset of the QQPPI network containing hubs and bottlenecks only.
(JPG)

Table S1
**Topological properties of** 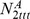 **.** The table contains all nodes, hubs and bottlenecks in 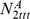 along with their topological properties according to tYNA.(XLSX)

Table S2
**Topological properties of** 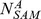 **.** The table contains all nodes, hubs and bottlenecks in 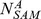 along with their topological properties according to tYNA.(XLSX)

Table S3
**Topological properties of** 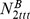 **.** The table contains all nodes, hubs and bottlenecks in 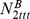 along with their topological properties according to tYNA.(XLSX)

Table S4
**Properties of nodes in core functional modules.** The table contains nodes in the core functional modules detected in 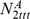, 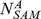 and 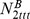 along with their degree, betweenness score and the number of their occurrences in 3- and 4-cliques.(XLSX)

Table S5
**Co-expression table for proteins interacting within the core functional module in** 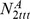 **.** This table contains the interactions within the core functional module in the network 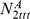, along with their Pearson correlation coefficients (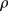) in control (C) and disease (D) samples, net difference of 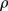 in control and disease samples (C–D) and their percentage of maximum possible change from control to disease, expressed as [{(C–D)/max(C–D)} * 100]. Here, max (C–D) is 2 as 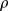 lies within the closed interval [−1, 1].(XLSX)

Table S6
**Co-expression table for proteins interacting within the core functional module in** 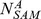 **.** This table contains the interactions within the core functional module in the network 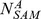, along with their Pearson correlation coefficients (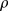) in control (C) and disease (D) samples, net difference of 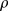 in control and disease samples (C–D) and their percentage of maximum possible change from control to disease, expressed as [{(C–D)/max(C–D)} * 100]. Here, max (C–D) is 2 as 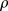 lies within the closed interval [−1, 1].(XLSX)

Table S7
**Co-expression table for proteins interacting within the core functional module in** 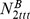 **.** This table contains the interactions within the core functional module in the network 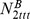, along with their Pearson correlation coefficients (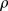) in control (C) and disease (D) samples, net difference of 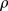 in control and disease samples (C–D) and their percentage of maximum possible change from control to disease, expressed as [{(C–D)/max(C–D)} * 100]. Here, max (C–D) is 2 as 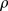 lies within the closed interval [−1, 1].(XLSX)

File S1 **Clique finding procedure.** The file contains the complete procedure, including the algorithm developed by us, which we have used to detect 3- and 4-cliques in the QQPPI networks.
(DOCX)

File S2 **Complex finding procedure.** The file contains the complete procedure, including the algorithm developed by us, which we used to detect complexes in the QQPPI networks.
(DOCX)

File S3 Connectivity and betweenness distribution of nodes in the QQPPI networks.
(DOCX)
