# Supplementary figures and images for: Construction and Analysis of the Protein-Protein Interaction Networks Based on Gene Expression Profiles of Parkinson's Disease
Source: PLoS One. 2014 Aug 29;9(8):e103047. doi: 10.1371/journal.pone.0103047 (PMC4149362; doi:10.1371/journal.pone.0103047)

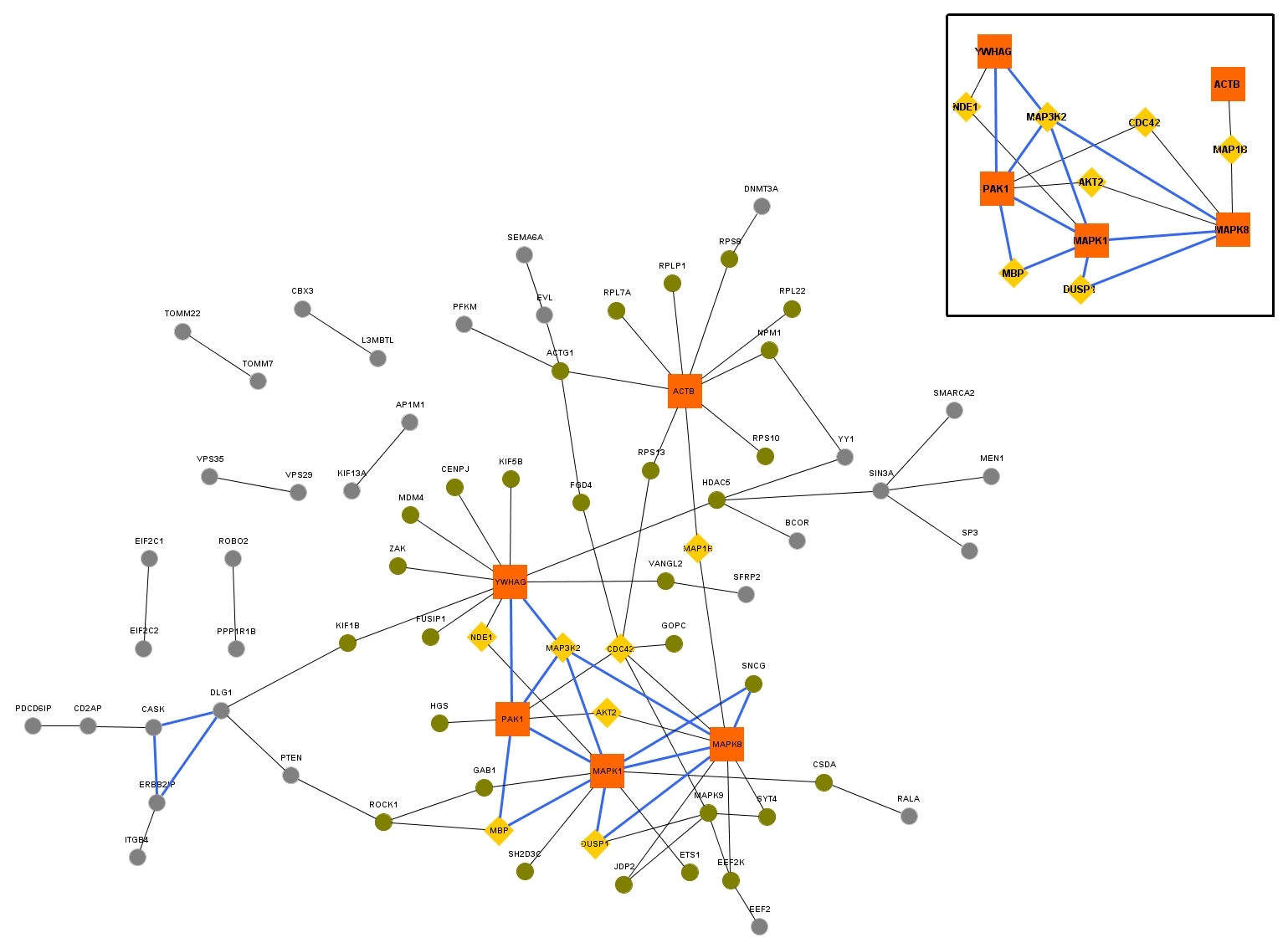

Supplement: Figure S1 — QQPPI network built from the dataset obtained using 2-tailed t -test (P<0.05) (GeneChip B). Orange coloured square nodes represent hubs (HC nodes). Yellow coloured triangular nodes represent bottlenecks (bottlenecks). The core functional module containing 3,4-cliques are represented using blue coloured edges. Non-hub non-bottleneck nodes are coloured green if they are directly connected to a hub or a bottleneck, and grey otherwise. Inset: Subset of the QQPPI network containing hubs and bottlenecks only. (JPG) [file pone.0103047.s001.jpg]
