# Supplementary material for: Construction and Analysis of the Protein-Protein Interaction Networks Based on Gene Expression Profiles of Parkinson's Disease
Source: PLoS One. 2014 Aug 29;9(8):e103047. doi: 10.1371/journal.pone.0103047 (PMC4149362; doi:10.1371/journal.pone.0103047)
Supplement: File S1 — Clique finding procedure. The file contains the complete procedure, including the algorithm developed by us, which we have used to detect 3- and 4-cliques in the QQPPI networks. (DOCX) [file pone.0103047.s009.docx]

**Supplementary file 1:**

The complete procedure for finding cliques is given as follows:

**Stage 1: Representing nodes in each edge with numbers:**

All the nodes(proteins) present in the network are sorted alphabetically and stored in a file “Nodes.txt”. The edges(interactions) are stored in another file “Edges.txt”.

The nodes in the file “Edges.txt” are converted to digits as per their order in the sorted file “Nodes.txt” and stored in file “Numeric.txt”. This conversion is done because it is easy to work with integers than with strings.

Example:

Nodes.txt Edges.txt Numeric.txt

AMPH AMPH CDK5 0 2

AP2A2 AP2A2 CDK5 1 2

CDK5 AP2A2 AMPH 1 0

**Stage 2: Finding 3 & 4-Cliques:**

After preparation of the required files, the following pseudo code is used for the required computation:

*1. Input : “Numeric.txt”*

*2. m= number of nodes in the network*

*3. n = number of edges in the network*

*4. x=0*

*5. for i=0 to n do*

*6. find all the nodes connected with node x and store in an array(int b[]).*

*7. l is the number of nodes connected with i^th^ node.*

*8. for k=0 to l do*

*9. p=b[k] < p is a node which is connected to x>*

*10. for j=0 to n do*

*11. for z= 0 to l <find the node connected with p, let q represent that node>*

*12. if q=b[z] then*

*13. Print x, p, q <form a 3-clique>*

*14. end if*

*15. end for z*

*16. end for j*

*17. end for k*

*18. reset l=0;*

*19. if x<m*

*20. increment x by 1*

*21. end for i*

The 4-cliques are also found in a similar way.
