# Supplementary material for: Construction and Analysis of the Protein-Protein Interaction Networks Based on Gene Expression Profiles of Parkinson's Disease
Source: PLoS One. 2014 Aug 29;9(8):e103047. doi: 10.1371/journal.pone.0103047 (PMC4149362; doi:10.1371/journal.pone.0103047)
Supplement: File S2 — Complex finding procedure. The file contains the complete procedure, including the algorithm developed by us, which we used to detect complexes in the QQPPI networks. (DOCX) [file pone.0103047.s010.docx]

**Supplementary file 2:**

The complete procedure for detecting complexes is given as follows:

**Stage 1: Designing the complex table:**

Each clique forming node(protein) is given as query protein in CORUM database and the ids of the complexes containing that protein are stored in an array format in the file “ComplexTable.txt”.

Example :

| AP2A2 | 46 | 148 | 247 | 970 | 1228 |
| --- | --- | --- | --- | --- | --- |
| CLTC | 567 | 2836 | 2837 | 5344 | 5345 |
| INSR | 1093 | 2577 | -1 | -1 | -1 |
| SNCA | 5830 | -1 | -1 | -1 | -1 |

here, the empty spaces are filled by -1.

**Stage 2: Finding complexes**

After preparation of the required files, the following pseudo code is used for the required computation:

*1. Input : “ComplexTable.txt”*

*2. m= number of genes*

*3. n= maximum number of complex associated with a single gene.(n=5 for above table)*

*4. a = array containing complex ids.*

*5. < Each id is taken one at a time and searched for its association with other proteins. Finally all the clique forming proteins associated with that id are detected as a complex. This process is repeated for all the ids>*

*6. for i=0 to m-1 do*

*7. for l=0 to n do*

*8. x=a[i][l]*

*9. if x=-1 then ignore*

*10. else*

*11. search for the presence of x in the rest of the array.*

*12. print all the nodes associated with id x as a complex.*

*13. end for l*

*14. end for i*
