# Supplementary material for: Construction and Analysis of the Protein-Protein Interaction Networks Based on Gene Expression Profiles of Parkinson's Disease
Source: PLoS One. 2014 Aug 29;9(8):e103047. doi: 10.1371/journal.pone.0103047 (PMC4149362; doi:10.1371/journal.pone.0103047)
Supplement: File S3 — Connectivity and betweenness distribution of nodes in the QQPPI networks. (DOCX) [file pone.0103047.s011.docx]

**S3.1: Connectivity distribution of nods in the QQPPI networks:**

**Figure S3.1.1: Connectivity distribution of nodes in** $\boldsymbol{N}_{\boldsymbol{2}\boldsymbol{ttt}}^{\boldsymbol{A}}$**:**

**Figure S3.1.2: Connectivity distribution of nodes in** $\boldsymbol{N}_{\boldsymbol{SAM}}^{\boldsymbol{A}}$**:**

**Figure S3.1.3: Connectivity distribution of nodes in** $\boldsymbol{N}_{\boldsymbol{2}\boldsymbol{ttt}}^{\boldsymbol{B}}$**:**

**S3.2: Betweenness distribution of nods in the QQPPI networks:**

**Figure S3.2.1: Betweenness distribution of nodes in** $\boldsymbol{N}_{\boldsymbol{2}\boldsymbol{ttt}}^{\boldsymbol{A}}$**:**

**Figure S3.2.2: Betweenness distribution of nodes in** $\boldsymbol{N}_{\boldsymbol{SAM}}^{\boldsymbol{A}}$**:**

**Figure S3.2.3: Betweenness distribution of nodes in** $\boldsymbol{N}_{\boldsymbol{2}\boldsymbol{ttt}}^{\boldsymbol{B}}$**:**
